# Integrating *RHD* Genotyping for More Accurate Rh(D) Antigen Phenotyping: A Retrospective Study

**DOI:** 10.3390/medicina61040670

**Published:** 2025-04-05

**Authors:** Mohammad Barouqa, Nestor Dela Cruz

**Affiliations:** Department of Pathology, University of South Alabama, Mobile, AL 36688, USA; ndelacruz@health.southalabama.edu

**Keywords:** Rh(D) antigen, *RHD* genotype, transfusion medicine, alloimmunization, transfusion, blood bank

## Abstract

*Background and Objectives:* The Rh blood group system is highly polymorphic, and accurate classification of Rh(D) variants is critical in transfusion medicine to prevent alloimmunization and optimize blood utilization. Despite the advances in conventional serologic testing, weak and partial Rh(D) phenotypes still remain challenges in Transfusion Medicine practice. The objective is to implement and assess the impact of *RHD* genotyping in classifying Rh(D) antigen status. *Materials and Methods:* We conducted a retrospective study at the University of South Alabama Medical Center and Children and Women’s Hospital between 1 January 2023 and 31 December 2024 to assess the impact of *RHD* genotyping in cases with discrepant Rh(D) typing, Rh(D)-positive patients with anti-Rh(D) antibodies, and neonates with positive weak Rh(D) tests. ABO and Rh(D) antigen typing was performed on 12,994 patients, including 3767 newly tested individuals. Weak Rh(D) testing was performed on newly tested individuals using automated microplate direct agglutination, followed by molecular genotyping. *Results:* Among the 25 patients with weak or discrepant Rh(D) phenotypes, weak Rh(D) variants were observed in 52% of cases, with Weak Type 2 being the most common, particularly in pediatric (age < 18 years old) patients. Partial Rh(D) phenotypes were identified in 40% of cases, predominantly among Black individuals. Three patients were reclassified as Rh(D)-positive based on genotyping and received 615 Rh(D)-positive RBC units without evidence of alloimmunization, while four patients were confirmed at risk of alloimmunization and remained classified as Rh(D)-negative. Fisher’s exact test demonstrated a significant association between ethnicity and Rh(D) classification (*p* < 0.01), and the McNemar exact test confirmed a significant reclassification of cases from Rh(D)-negative to Rh(D)-positive (*p* < 0.01). *Conclusions: RHD* genotyping enhances the accuracy of Rh(D) antigen classification, mitigating alloimmunization risks and the unnecessary use of Rh Immunoglobulin and optimizing blood product utilization. The reclassification of patients to Rh(D)-positive alleviates pressure on Rh(D)-negative blood supplies, particularly during critical shortages. These findings underscore the necessity of integrating molecular *RHD* testing into routine transfusion medicine practices to improve patient safety and resource management.

## 1. Introduction

The Rh (formerly known as Rhesus) blood group is an extensively immunogenic and polymorphic group of antigens expressed on red blood cells and incorporated into many clinical complications related to their antibodies, especially as they can cause acute hemolytic transfusion reactions, delayed hemolytic transfusion reactions, and hemolytic disease of fetuses and newborns (HDFN) [1,2]. The expression of Rh antigens (the Rh(D) antigen, C antigen, c antigen, E antigen, and e antigen) stems from the *RHD* and *RHCE* genes located on chromosome 1 (1p34-p36) [1]. In addition to the five main Rh antigens expressed and traced by transfusion medicine services, there are at least 50 other Rh antigens defined and resulting from either point mutations, hybrid alleles, or both [3]. Blood transfusion and pregnancy are two well-established risk factors or events for alloimmunization, especially for Rh(D) antigens [4].

The expression of the Rh(D) antigen varies and mainly depends on serologic studies, which classified the expression into groups: Rh(D)-negative, weak Rh(D), partial Rh(D), DEL Rh(D), and the conventional or wild-type. In Rh(D)-negative Caucasians, gene deletion is the main reason for the absence of Rh(D) antigen expression, while in Black populations, the *RHD* pseudogene is the main mechanism behind the loss of Rh(D) expression [1]. In Asian populations, Rh(D) DEL or Rh(D) Elute(Del) is commonly reported and characterized by weak antigenic expression, with a prevalence of 30% among Asian Rh(D)-negative donors [5]. The weak Rh(D) phenotype is characterized by weak expression and reactivity (≤2+) with anti-Rh(D) reagents in initial tests but stronger agglutination with antihuman globulin, causing patients to type as Rh(D)-negative in routine testing.

The prevalence of weak Rh(D) phenotypes ranges from 0.2–1.0% among Caucasians in Europe and the United States, with most cases linked to weak Rh(D) types 1, 2, and 3 [6]. Patients with weak types 1, 2, and 3 (either in homozygous or hemizygous states) express Rh(D) epitopes at a decreased density. Hence, patients are not considered at risk of developing anti-Rh(D) antibodies and can be managed as Rh(D)-positive, which eliminates the need for Rh(D) immune globulin (RhIG) prophylaxis in pregnant females. However, other weak Rh(D) types, such as weak Rh(D) 4.2 (DAR), 11, 15, 21, and 57, have been associated with cases of alloimmunization, and patients with these subtypes are managed as Rh(D)-negative [6]. In partial Rh(D) phenotypes, the absence of specific epitope expression on the Rh(D) antigen put patients receiving Rh(D)-positive units or the delivery of Rh(D)-positive newborns at risk of alloimmunization. In these partial Rh(D) phenotypes, RBCs may agglutinate during immediate spin testing with anti-Rh(D) reagents and are often interpreted as Rh(D)-positive [7].

The most common partial Rh(D) phenotypes in Europe are DNB, DVI, and DVII, while in the United States, most individuals with partial Rh(D) phenotypes are of African ancestry [7,8]. In Transfusion Medicine practice, patients with partial Rh(D) phenotypes are typed as Rh(D)-negative to prevent alloimmunization for transfusion purposes and as Rh(D)-positive for blood donation purposes [6]. DEL (Del) phenotypes appear as Rh(D)-negative when conventional blood testing is applied and require adsorption and elution techniques for detection, with RHD*DEL1 (RHD:c.1227G > A allele) being the most prevalent DEL in East Asia [9]. Although there have been very few cases reported with Asian DEL in the last three decades, the current recommendation is treating patients with Asian-type DEL as Rh(D)-positive for transfusion and Rh immune prophylaxis purposes [10]. Furthermore, recent molecular studies performed to assess the Rh(D) antigen have also shown a variant known as D *DAU0, which encodes one amino acid change (Thr379Met) and does not appear to be missing any epitopes or have weak antigenic expression [11].

In Transfusion Medicine, donor blood is tested using methods that detect weak Rh(D) antigen expression on red blood cells as these units must be labeled as Rh(D)-positive. However, weak Rh(D) testing is generally unnecessary for patients receiving blood, except in specific cases, such as assessing the red cells of an infant whose mother is at risk of Rh(D) alloimmunization or if Rh(D) discrepancy is detected. In these cases, weak Rh(D) testing is typically performed using Indirect Antiglobulin Testing (IAT) to determine whether the red cells have any Rh(D) antigen expression on their surface [12].

The introduction of FDA-licensed monoclonal IgM reagents has simplified Rh(D) typing. These reagents identify many samples as Rh(D)-positive (where IAT testing was previously required) while accurately typing partial DVI subtypes as Rh(D)-negative [13]. This advancement has addressed and helped in classifying patients with Rh(D) variants as D-positive. Despite these improvements in serologic typing, Rh(D) typing discrepancies still remain a challenge, requiring some patients to receive Rh(D)-negative units until the discrepancies are resolved, in addition to Rh(D)-positive patients with anti-Rh(D) antibodies that continue to be a common challenge in Transfusion Medicine. Furthermore, in obstetrics, neonates born to Rh(D)-negative mothers undergo serologic testing for weak Rh(D) in order to determine if the mother would need RhIG to provide protection from further alloimmunization. This serologically weak Rh(D) testing does not differentiate between weak and partial Rh(D) antigens [13], necessitating the selection and application of the appropriate *RHD* genotyping (low-, medium-, or high-resolution) for accurate differentiation [14].

The clinical impact and laboratory application of genotyping *RHD* to classify the Rh(D) antigen after performing weak Rh(D) testing have not been extensively studied in the US and Canada [15]. Furthermore, in 2024, the United States faced significant blood shortages, with the American Red Cross reporting the lowest donor turnout in two decades. This decline was exacerbated in July 2024, which led to a 25% drop in the national blood inventory. Additionally, disruptions in the Southeast, including cyberattacks and environmental factors like hurricanes and storms, further strained the national blood supply [16].

In this manuscript, we sought to implement and assess the impact of *RHD* genotyping in our laboratory for cases with discrepant Rh(D) typing, Rh(D)-positive patients with anti-Rh(D), and newborns with positive weak Rh(D) tests.

## 2. Materials and Methods

This retrospective study was approved by the University of South Alabama Institutional Review Board (IRB protocol: 24–550) in Mobile, Alabama on 5 December 2024, and a waiver of subject authorization was properly granted prior to data collection. The data were collected anonymously and serially coded to ensure confidentiality.

We reviewed all the testing performed in the laboratories on patients from the period of 1 January 2023 to 31 December 2024. The review included initial serologic ABO/Rh(D), serologic weak Rh(D), and Rh(D) genotype tests, followed by Rh(D) genotyping on patients with positive serologic weak Rh(D) test results, Rh(D)-positive individuals with anti-Rh(D) antibodies, and neonates born to Rh(D)-negative mothers who tested positive for weak Rh(D).

**Figure 1:**
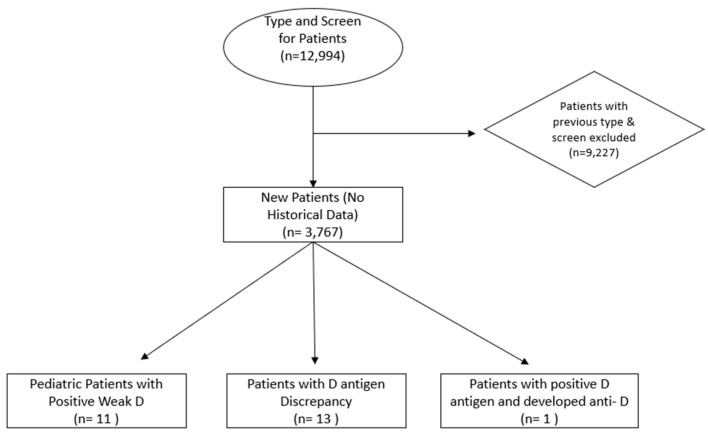
Selection and exclusion process of patients. Typing and screening performed via serologic testing.

The total number of patients who underwent ABO and Rh(D) antigen typing was 12,994. Patients with prior typing and screening (tested by serology and/or genotyping) that established ABO/ Rh(D) type and had no discrepancies or developed anti-Rh(D) (*n* = 9227) were excluded, leaving 3767 new patients with no historical data or showing discrepant results. Serologic weak Rh(D) antigen testing was performed on the latter group who initially tested negative for Rh(D) to confirm that there was no weak or partial Rh(D) expression. *RHD* genotyping was performed on patients who tested positive for the serologic weak Rh(D) antigen or were found to have anti-Rh(D) and were historically typed as Rh(D)-positive. These patients were then categorized into three groups based on serological findings: pediatric patients with positive weak Rh(D) (*n* = 11), patients with Rh(D) antigen discrepancy (*n* = 13), and patients with the positive Rh(D) antigen who developed anti-Rh(D) (Figure 1).

Testing was performed at the University of South Alabama Medical Center, a combined academic and trauma center in Mobile, Alabama, and at the Children and Women’s Hospital, a specialized academic and medical facility also located in Mobile, Alabama. The laboratories performed all testing as per the manufacturer’s directions. The initial typing for the Rh(D) antigen was performed in automated microplate direct agglutination (MDA) using two monoclonal IgM reagents in Series 4 (D4, clone MS201) and Series 5 (D5, TH28) using (Echo^®^ Lumena, Immucor, Norcross, GA, USA). First-time patients were confirmed using manual Rh(D) typing (D4, Clone MS201, Immucor, Norcross, GA, USA). Weak Rh(D) testing was performed using IgG and IgM reagents in automated MDA (Echo^®^, Lumena, Immucor, Norcross, GA, USA). Samples that required molecular testing were sent to a reference laboratory (National Molecular Laboratory, American Red Cross, Philadelphia, PA, USA) that isolated genomic DNA from peripheral white blood cells using the QIAamp DNA Mini kit (QIAGEN, Carlsbad, CA, USA). Genomic DNA was analyzed using RHD BeadChip™ (Werfen, Norcross, GA, USA). QC was performed as per the laboratory’s policies and the vendor package insert using WB corQC (Echo^®^, Lumena, Werfen, Norcross, GA, USA). 

Statistical analysis was performed in R studio (V.2023.12.0) and included a Fisher’s exact test with the Freeman–Halton extension for categorical analysis of ethnicity vs. D classification. Furthermore, the Mcnemar exact test was used to assess if genotype testing leads to a significant reclassification after considering the size of the cohort that underwent *RHD* genotyping [17]. The threshold for statistical significance was set at *p*-value < 0.05.

## 3. Results

Over the span of 2 years, we identified 25 patients with weak or discrepant Rh(D) phenotypes with ages ranging from 1 day to 63 years old and required *RHD* variant genotyping to reclassify their Rh(D) antigen.

The cohort included 12 (48%) male patients and 13 (52%) female patients. Five cases (20%) were obstetrical cases for either pregnant patients establishing prenatal care or transferred to our facility to deliver. Nineteen cases (76.0%) were non-obstetrical cases, which included 11 (44%) pediatric patients (defined as patients younger than 18 years old) born with weak Rh(D) and who required molecular testing to further characterize the Rh(D) antigen expressed on their red blood cells. Four (16%) cases were sickle-cell patients who were receiving red cell exchanges, and the remaining were for oncology patients who were transfusion-dependent due to their chemotherapy regimen. Fourteen (56%) cases were typed as ABO blood group “O”, and only one (4%) case was found to have an antibody to the Rh(D) antigen (anti-Rh(D)) due to a partial Rh(D) antigen (Allele RHD08N.01/ RHD*09.02.01).

The number of patients that required red blood cell transfusions was seven (28%), including the sickle-cell patients. The total and mean number of red blood cell units transfused in the studied cohort were 1051 and 150 units, respectively.

The number of patients who were switched to Rh(D)-positive was 3 patients, who received a total of 615 Rh(D)-positive RBC units based on their *RHD* genotype, while the other 4 patients were found at risk of Rh(D) alloimmunization, kept as Rh(D)-negative and received a total of 436 RBC units. Weak Rh(D) types were seen in 13 (52%) cases studied in the cohort, with the most common being Weak Rh(D) Type 2, observed predominately in 54% of pediatric patients and approximately equal to Weak Type 1 in adult patients (Table 1).

Weak Rh(D) was predominantly detected in Caucasian populations. Partial D variants were detected in 10 (40%) of the patients studied and in 16% of pediatric patients. Partial variants were frequently found among black individuals, with variants like RHD*DIIIa-CE(4-7)-D and RHD*09 showing a combined prevalence of 6% across all groups. Conventional Rh(D) variants were detected in two cases (9%) that were tested for molecular antigenic expression, which included D variant testing. Notably, Asian representation is minimal across all variant types with one (4%) case tested showing weak D variant type 2 (Table 2)

Fisher’s exact test with the Freeman–Halton extension was used to assess the relationship between ethnicity and Rh(D) classification ((conventional Rh(D), equivalent or weak type 1 or 2) vs. partial) and was significant with a *p*-value < 0.01 (Table A1). One case included the *Rh(D) DAU0* allele and was reclassified as Rh(D)-positive as *DAU* variants are equivalent to the conventional Rh(D) antigen. The Mcnemar exact test was used to assess the reclassification of cases from initially Rh(D)-negative to Rh(D)-positive and was significant with a *p*-value < 0.01 (Table A2).

## 4. Discussion

This study emphasizes the critical role of *RHD* genotyping in resolving discrepancies in serologic Rh(D) typing and guiding transfusion and immunoprophylaxis. It also discusses an important and powerful tool to further characterize the Rh(D) antigen status of patients and exclude the limitations caused by serologic testing, especially when patients expected to receive multiple blood units at a time of national shortage is an enormous limiting step. Previous studies have highlighted the limitations of serologic testing in accurately distinguishing weak and partial Rh(D) phenotypes, leading to misclassification and potential alloimmunization [7,18]. Our findings demonstrate that the application of molecular techniques can significantly improve the precision of Rh(D) classification, allowing antigenic reclassification, which will aid in reducing unnecessary RhIG use in obstetric patients, providing the correct Rh(D) type for neonates, and helping Transfusion Medicine services to maintain their inventories.

Traditional serologic testing has limitations in distinguishing between weak and partial Rh(D) phenotypes, which can lead to the misclassification of the Rh(D) status of patients, the unnecessary use of RhIG, and alloimmunization [18]. FDA-licensed monoclonal IgM reagents have improved the accuracy of Rh(D) typing but do not eliminate the need for *RHD* genotyping in cases with typing discrepancies or weak antigenic expression [13].

Over a two-year period, we identified 25 patients with weak or discrepant Rh(D) phenotypes, spanning a diverse cohort of pediatric, obstetric, and transfusion-dependent patients due to sickle cell disease. Weak Rh(D) phenotypes, particularly types 1 and 2, were the most frequently observed and predominantly identified in Caucasian populations [19]. These variants are well-documented as not posing a risk for anti-Rh(D) alloimmunization, and our data support and align with the previously reported classification of these patients as Rh(D)-positive [14]. Conversely, partial Rh(D) phenotypes were more prevalent among Black individuals, consistent with prior studies [20]. These patients, who lack specific epitopes, were appropriately classified as Rh(D)-negative to mitigate the risk of alloimmunization either by blood transfusion and/or pregnancy.

The ability to differentiate between weak and partial Rh(D) phenotypes has profound implications for clinical practice. Weak Rh(D) types 1, 2, and 3—when identified through genotyping—can be confidently reclassified as Rh(D)-positive, enabling the use of Rh(D)-positive blood units and conserving the limited supply of Rh(D)-negative units. This approach was evident in our study, where three patients were reclassified as Rh(D)-positive and safely received 615 Rh(D)-positive RBC units without evidence of alloimmunization. Such reclassification has the dual benefit of ensuring patient safety while addressing the ongoing national blood shortages and aligns with previously published studies that adopted a similar approach in reclassifying patients’ Rh(D) phenotypes using molecular testing [21].

Partial Rh(D) phenotypes, on the other hand, pose a significant alloimmunization risk when exposed to Rh(D)-positive blood or during pregnancy with a Rh(D)-positive fetus. Our identification of partial Rh(D) variants, such as *RHDDIIIa-CE(4-7)-D and RHD09*, underscores the necessity of maintaining these patients as Rh(D)-negative for transfusion purposes. This distinction is crucial in preventing alloimmunization events in obstetric and non-obstetric patients.

Our findings align with the well-established ethnic variability in Rh(D) antigen expression where weak Rh(D) phenotypes were predominantly observed in Caucasian patients, while partial Rh(D) variants were more common in Black individuals [3]. Asian representation was minimal in our cohort, which can be attributed to the population demographic. Fisher’s exact test with the Freeman–Halton extension demonstrated a significant association between ethnicity and Rh(D) classification as weak vs. partial (*p* < 0.01). These observations highlight the importance of considering these variations in Rh(D) expression when implementing genotype testing, especially for blood donors, as it has been reported that exposure to altered Rh(D) antigens from specific ethnicities of donors can lead to the alloimmunization of blood recipients that carry conventional *RHD* or *DAU0* [11]. The association between ethnicity (Caucasian vs. non-Caucasian) and RhD-negative status after reclassifying patients according to their molecular results was assessed using an odds ratio (OR) with a 95% confidence interval (CI), which revealed that the odds of being reclassified as RhD-negative were significantly lower among Caucasians compared to non-Caucasians, with an odds ratio of 0.042 (95% CI: 0.004, 0.444).

Although the number of patients in our cohort is small, using the McNemar exact test revealed a significant reclassification of cases from Rh(D)-negative to Rh(D)-positive (*p* < 0.01), affirming the added accuracy provided by *RHD* genotyping, and that a substantial number of cases were reclassified after performing genotype tests. This significant reclassification has direct implications for addressing blood shortages because by reclassifying patients who would serologically type as Rh(D)-negative to receive Rh(D)-positive blood, genotyping alleviates the strain on Rh(D)-negative blood supplies, especially for ABO blood group “O”. This operational benefit is particularly crucial in the current landscape of critical blood shortages.

Our study is limited by its single-center design and relatively small sample size. Furthermore, the minimal representation of Asian individuals limits our ability to reach broader conclusions about specific variants including the DEL phenotypes in this population, which have been reported at higher frequencies in East Asian populations [22]. While we acknowledge this limitation, it is important to note that the small size is a common challenge in rare antigenic phenotypes. Future multicenter studies with larger, more diverse cohorts and preferably defined by geographical area are needed to further elucidate the clinical and testing benefits of *RHD* genotyping. Molecular testing is indeed a powerful tool for identifying Rh(D) variants, but it is not without its challenges. The accuracy of genotyping is dependent on the quality of the DNA samples, and there is always a risk of misclassification due to sample handling, contamination, or DNA degradation. Furthermore, as our knowledge about the Rh(D) variant expands and new variants are identified, there is always a limitation that certain Rh(D) variants may not be covered by the currently available testing panels.

## 5. Conclusions

*RHD* genotyping represents a significant and transformative approach in Transfusion Medicine, enhancing the precision of transfusion practices and supporting the optimal utilization of Rh(D)-negative blood products. The integration of molecular techniques into routine laboratory and blood bank workflows should be considered a priority, particularly in the context of ongoing blood shortages and the increasing complexity of patient populations served by Transfusion Medicine. Furthermore, it allows the avoidance of unnecessary RhIG use for obstetric patients. Factors such as the cost of molecular testing, the availability of necessary technology, and the need for staff training must be carefully considered in addition to integrating and establishing the testing into the correct context.

## Figures and Tables

**Table 1:** Distribution and management of D variants across age groups, blood types, and clinical contexts.

*RHD* Alleles	Rh(D) Type (Based on RHD Variant)	Pediatric (Age < 18 Years Old)	Adult	ABO Blood Group “O”	Rh(D) Management (Switched to Rh(D) Positive	Obstetrics	Non-Obstetrics
RHD*01W.1 (homozygous)	Weak Type 1	2	4	2	Y	4	2
RHD*01W.2 (RHD*weak D type 2) (homozygous)	Weak Type 2	4	3	4	Y	1	6
Conventional D *01	Conventional	1	1	2	Y	0	2
RHD*09(RHD*DAR)/RHD*DIIIa-CE (4–7)-D	Partial D, Altered C	1	1	2	N	0	2
RHD*09.03 (homozygous)	Weak partial D type 4	1	0	1	N	0	1
RHD*60/RHD*08N.01	Partial D	0	1	1	N	0	1
RHD*DIIIa-CE(4-7)-D (homozygous)	D-altered C+	1	1	0	N	0	2
RHD*DIIIa (homozygous)	Partial D+	0	1	1	N	0	1
RHD*DVI (homozygous)	Partial D	1	0	0	N	0	1
RHD08N.01/RHD*09.02.01	partial D DAR2	0	1	1	N	0	1
RHD*08N.01/ RHD*DAU0	Conventional D	0	1	1	Y	0	1

**Table 2:** Distribution of D variants by age, ethnicity, and Rh(D) antigen classification.

*RHD* Alleles	Rh(D) Type (Based on *RHD* Variant)	Pediatric (Age < 18 Years Old)	Adult	Caucasian	Black	Asian	Rh(D) Antigen Classification
RHD*01W.1 (homozygous)	Weak Type 1	2	4	6	0	0	Weak
RHD*01W.2 (RHD*weak D type 2) (homozygous)	Weak Type 2	4	3	5	1	1	Weak
Conventional D *01	Conventional	1	1	1	1	0	Conventional
RHD*09(RHD*DAR)/RHD*DIIIa-CE (4-7)-D	Partial D, Altered C	1	1	0	2	0	Partial
RHD*09.03 (homozygous)	Weak partial D type 4	1	0	0	1	0	Partial
RHD*60/RHD*08N.01	Partial D	0	1	0	1	0	Partial
RHD*DIIIa-CE(4-7)-D (homozygous)	D-altered C+	1	1	0	2	0	Partial
RHD*DIIIa (homozygous)	Partial D+	0	1	0	1	0	Partial
RHD*DVI (homozygous)	Partial D	1	0	1	0	0	Partial
RHD08N.01/RHD*09.02.01	partial D DAR2	0	1	0	1	0	Partial
RHD*08N.01/RHD*DAU0	Conventional D	0	1	0	1	0	Partial

## Data Availability

The de-identified datasets analyzed during this study are available from the corresponding author upon reasonable request.

## Abbreviations

RhIG: Rh(D) immune globulin

## Appendix A

**Table A1:** Contingency table for Fisher’s exact test with Freeman–Halton extension.

Rh(D) Classification	Caucasian	Black	Asian	Total
Weak	11	1	1	13
Conventional	1	2	0	3
Partial	2	7	0	9
Total	14	10	1	25

*p*-value: 1.40 × 10^−11^.

**Table A2:** Contingency table for Mcnemar exact test.

Before to After	Y (Changed to Y)	N (No Change)	Total
N (Before N)	16	9	25
Y (Before: Y)	0	0	0

*p*-value: 3.05 × 10^−5^.
